# A Digital Single-Session Intervention Platform for Youth Mental Health: Cultural Adaptation, Evaluation, and Dissemination

**DOI:** 10.2196/43062

**Published:** 2023-02-14

**Authors:** Akash Shroff, Chantelle Roulston, Julia Fassler, Nicole A Dierschke, Jennifer San Pedro Todd, Ámbar Ríos-Herrera, Kristen A Plastino, Jessica Lee Schleider

**Affiliations:** 1 Department of Psychology Stony Brook University Stony Brook, NY United States; 2 University of Texas Teen Health University of Texas Health Science Center at San Antonio San Antonio, TX United States

**Keywords:** single-session intervention, cultural adaptation, web-based intervention, adolescents, mobile phone

## Abstract

**Background:**

Despite the proliferation of evidence-based digital mental health programs for young people, their low uptake and inconsistent implementation preclude them from benefiting youths at scale. Identifying effective implementation strategies for evidence-based supports is especially critical in regions where treatment access is lowest owing to mental health provider shortages.

**Objective:**

The goal of this academic-community partnership, funded by the City of San Antonio Metropolitan Health District, was to culturally adapt, disseminate, and gauge the acceptability and utility of an evidence-based digital mental health platform—Project Youth Empowerment and Support (YES)—among English- and Spanish-speaking youths living in south Texas.

**Methods:**

Project YES is an open-access, anonymous platform containing 3 evidence-based, self-guided interventions for youth mental health. Project YES was culturally adapted via focus groups and co-design sessions with San Antonio youths with lived experience of depression and anxiety; translated into Spanish; and disseminated throughout San Antonio, Texas, via community and school partnerships.

**Results:**

During the project period (April 2021 to December 2021), 1801 San Antonio youths began and 894 (49.64%) of them completed a 30-minute, single-session intervention within Project YES (aged 11-17 years; n=718, 39.87% male; n=961, 53.36% female; and n=3, 0.17% intersex; n=1477, 82.01% Hispanic; n=77, 4.28% non-Hispanic White; n=113, 6.27% Black; n=28, 1.55% Asian; and n=93, 5.16% other). This completion rate (49.64%) surpassed those previously observed for Project YES (eg, 34% when disseminated via social media). San Antonio youths rated Project YES as highly acceptable across all metrics, both in English and Spanish. In addition, the youths who completed Project YES—ENGLISH reported significant improvements in hopelessness (Cohen *d*=0.33; *P*<001), self-hate (Cohen *d*=0.27; *P*<001), and perceived agency (Cohen *d*=0.25; *P*<001) from before to after the intervention, and the youths who completed Project YES—SPANISH reported significant improvements in self-hate (Cohen *d*=0.37; *P*=.049) from before to after the intervention.

**Conclusions:**

The results indicate that Project YES—an open-access, free, and anonymous web-based single-session intervention platform—is an acceptable, accessible, and applicable mental health support for English- and Spanish-speaking San Antonio youths.

## Introduction

### Background

Most adolescents with mental health needs in the United States are unable to access evidence-based interventions when and where they are needed [1]. This need-to-access gap has remained stagnant for decades because of a myriad of factors, including provider shortages nationwide; a dearth of therapists trained in evidence-based approaches; premature dropouts from multisession treatments; cost and transportation challenges; and language barriers, as many evidence-based treatments are accessible only to English-speakers [2-4]. All these access barriers grew starker with the onset of the COVID-19 pandemic when public health mandates and social distancing precautions further limited access to face-to-face treatment options [5]. Given these compounding factors, interest has dramatically grown in digital, self-guided mental health supports (ie, those accessible via computers and smartphones, which are accessible to >95% of the US adolescents) [6], which hold the potential to be more accessible and scalable than in-person interventions. However, the real-world uptake and completion rates of self-guided digital interventions are exceptionally low, on average [7]; many clinically tested digital self-help tools are never made openly accessible [8], few evidence-based self-help tools are available in languages other than English, and popular mental health apps often include no elements of evidence-based interventions [9]. Thus, there is a pressing need for digital youth mental health supports that are simultaneously evidence-based, easily accessible to diverse youths in moments of need, and optimized for uptake and completion in community settings.

Digital, evidence-based single-session interventions (SSIs) may help address the need for accessible, easily completable, and effective mental health interventions for adolescents. SSIs are defined as “structured programs that intentionally involve only one visit or encounter with a clinic, provider, or program; they may serve as stand-alone or adjunctive clinical services” [10]. SSIs can take multiple forms (eg, provider delivered and self-guided); however, the flexible and scalable nature of *digital, self-administered* SSIs allows them to be delivered and completed in low-resource settings relatively rapidly and broadly. Existing literature supports the effectiveness of SSIs for adolescents’ depression and anxiety, even when delivered as stand-alone mental health supports [11-14]. Furthermore, in a recent nationwide randomized trial including 2452 adolescents with elevated depressive symptoms, 2 different digital, self-guided SSIs (one teaching behavioral activation and another teaching that personal characteristics are malleable) significantly reduced 3-month depressive symptoms, hopelessness, and restrictive eating compared with a supportive control [15]. In another randomized, placebo-controlled trial of 555 adolescents endorsing self-injurious behaviors, a digital, self-guided SSI significantly reduced self-hatred and increased the desire to stop future nonsuicidal self-injury [16]. In both trials, adolescents completed the digital SSIs in the midst of the COVID-19 pandemic (between May and December 2020), and more than 80% of the adolescents who began a digital SSI completed it in full. Even when evaluated in naturalistic settings, where the completion rates for digital self-help tools drop as low as 1% (vs 44%-99% in paid research studies) [7], 34% of US adolescents completed digital mental health–focused SSIs that were publicly and freely accessible on the web [17]. When comparing the engagement rates of digital SSIs in randomized controlled trials versus naturalistic evaluations, the presence of participant payments emerged as the sole predictor of higher versus lower rates of SSI completion (neither the level of distress nor sexual orientation, race, ethnicity, and gender identity predicted differential completion rates) [17]. Nonetheless, even in the absence of monetary incentives, SSIs show feasibility and clinical utility. The completion rates for digital SSIs substantially surpass those seen for previously studied digital self-help tools in formal trials and naturalistic contexts alike.

Given their clinical utility, high completion rates, and low cost (the abovementioned SSIs are free and available on the web) and because youths increasingly turn to digital tools such as websites and apps for mental health information and support [18], SSIs represent a promising approach to supporting youths’ in-the-moment mental health needs. However, many questions remain regarding the dissemination and implementation of the existing SSIs within underserved communities. To date, no evidence-based SSIs have been systematically adapted to the culture, language, or context of specific underresourced and underserved populations—although such adaptations may both facilitate successful implementation and minimize the unintended consequences of failing to center population-specific needs (ie, inadvertently worsening treatment access disparities by disseminating English-only digital self-help tools in communities with many monolingual Spanish speakers). Cultural adaptations of interventions have demonstrated increased acceptability and retention in marginalized populations by including their stories and translations by talking to clinicians well versed in the cultural differences of specific populations [19,20]. However, startlingly, only a few youth-focused intervention trials—even those that include youths of color—actually use strategies to address culture within the interventions themselves [21]. Furthermore, very few culturally adapted interventions for adolescent anxiety and depression have been developed; those that exist rely mainly on clinician feedback, instead of youth involvement, to drive cultural adaptations [21]. This reflects an important and critical gap in the available digital supports.

Culturally adapted digital tools may be especially useful for low-resourced or minoritized communities because individuals belonging to these communities often rely on digital resources for health-related information [22]. A digital intervention app created for Spanish-speaking adults showed greater retention rates (72%) than most digital mental health apps. A pilot study testing this app demonstrated a preliminary reduction in depression severity and greater acceptability in the Spanish-speaking study population [23]. However, the use of digital interventions in these minoritized populations *without* careful adaptation can potentially have unintended negative consequences, such as alienating users with marginalized identities [22], making it crucial to rely on user and stakeholder feedback throughout the adaptation process. Overall, few evidence-based mental health interventions have been systematically adapted for specific minoritized or marginalized youth communities, and even when those adaptations *have* been deployed, youth feedback and perspectives on the interventions have rarely been formally incorporated (eg, via user-centered design approaches) [24]. Thus, efforts to culturally adapt youth-focused SSIs will need to proactively center on end users’ perspectives and experiences.

Latino, Latina, or Latine young people are a population with substantial and unmet mental health needs for whom culturally tailored digital mental health supports (including SSIs) may carry value. Latino, Latina, or Latine individuals are the largest ethnic minority in the United States, accounting for 18.5% of the US population [23]. Up to 13 million Latine individuals meet the criteria for a mental illness; however only 9.6% of these individuals access any evidence-based mental health support in a given year [24]. Barriers to treatment access vary widely, from limited insurance coverage to legal status, stigma, and language barriers (up to 33% Latine individuals report speaking English less than “very well” [25]); together, these create a clear need for scalable mental health tools tailored to the needs of Latine and Spanish-speaking individuals. Not only do most Latine and Spanish-speaking individuals in the United States have internet access via mobile devices (up to 80%), but a large majority also endorse openness to using digital health supports [26]. In addition, there is some evidence that Spanish-translated digital mental health tools are effective (3 studies investigating Spanish-language apps reported decreases in the target mental health scores such as depression or substance abuse [27]). However, few such tools have been built and evaluated to date [27]. Accordingly, the creation of translated and culturally relevant digital mental health supports is a critical next step for digital and SSI research.

Importantly, both Spanish- and English-speaking communities across different regions of the United States are highly diverse in themselves, and as such, taking a *community-* or *region-level* approach to adapting digital mental health tools may yield supports that are particularly acceptable to (and likely to be used by) specific communities. A community that may benefit from such efforts is that of Texas, one of the largest states in the United States, which is ranked by Mental Health America as among America’s worst-performing states when it comes to treatment access. For instance, among the youths in Texas with a major depressive episode last year, >73% did not access any form of mental health treatment—this was the worst percentage among all the states last year [28]. Specifically, San Antonio, Texas, and the surrounding area are designated as a high-need, low-income mental health professional shortage area by the Health Resources and Services Administration [29]. In addition, of all the people in San Antonio, approximately 57% speak only English and 39% speak only Spanish [30]. Therefore, accessible, culturally tailored, digital, and brief mental health supports are critically needed to help youths in San Antonio, Texas, manage their mental health needs flexibly, affordably, and in real-time moments of need. Adapting and evaluating digital, evidence-based SSIs for youths in this city promises to increase the adolescents’ odds of accessing *any* form of evidence-based mental health support, given that many San Antonio youths with mental health needs are currently accessing no support at all.

Accordingly, we led an academic-community partnership project to systematically adapt, translate, and disseminate digital SSIs for use by youths in the high-need city of San Antonio, Texas. In collaboration with youth stakeholders and local health care providers, we adapted an evidence-based single-session web-based intervention platform [31] and tested its acceptability, feasibility, and utility when disseminated as a city-wide resource to English- and Spanish-speaking young people in San Antonio. Our approach to cultural adaptation fits with the core principles of Heim and Kohrt’s [32] Cultural Adaptation Framework for Scalable Interventions, which centers on the integration of the *cultural concepts of distress* (here, reflected within San Antonio youths’ lived experience narratives and descriptions of their own difficulties and coping strategies)*,* along with adaptations to nonspecific intervention factors to increase cultural relevance (eg, the presence of a Spanish-translated version, youth feedback–informed language adaptations, and the inclusion of San Antonio youth voices throughout all programs). The specific platform adapted for this nonexperimental, observational project is called Project Youth Empowerment and Support (YES), which is a free-of-charge, open, and anonymous website wherein young people can anonymously and flexibly complete any of 3 digital SSIs, which have demonstrated short- and long-term effectiveness in reducing hopelessness, self-hate, and depression symptoms [12,15,31]. The 3 SSIs that adolescents may complete within Project YES are called “Project Personality” (which teaches that personal traits are malleable), “The ABC Project” (which teaches values-based activity engagement to elicit pleasure and accomplishment), and “Project CARE” (which teaches the benefits of self-kindness in social and academic success). These 30-minute SSIs were designed to instill adaptive self-relevant beliefs with known links to lower levels of depression and anxiety symptoms. The SSIs within Project YES have shown acceptability and both short- and long-term utility in reducing hopelessness, increasing agency, and mitigating depression and anxiety symptoms in young people across randomized and open trials alike [12,15,17,33]. Each SSI was created per a routinely used SSI design framework, which is detailed elsewhere [10]. Youths (1) learn the brain science that normalizes a core concept, (2) are invited to help researchers learn about their perspectives as “youth experts,” (3) are asked to convey the program’s messages in their own words and offer advice to their peers, and (4) hear stories from peers who used the program in their lives. Regardless of program selection, youths can choose to offer their “best, anonymous coping advice” to other youths coping with depression or anxiety. Youths are also given the opportunity to share this advice in a public “YES advice center” [10].

### Goal of This Study

In this project, the 3 digital SSIs in Project YES were adapted and revised in collaboration with youth stakeholders, translated into Spanish, and disseminated to youths across San Antonio, Texas, via collaborations with community organizations, clinics, and schools in the city. For both the English and Spanish versions of the interventions, we investigated youths’ acceptability of the programs and immediate effects on proximal, clinically relevant outcomes that the SSIs were designed to target (hopelessness, agency, perceived control, and self-hate). We examined use-pattern variables (eg, SSI selection and SSI completion rates), user characteristics (age range, sex, gender identity, race and ethnicity, and depressive symptoms), and acceptability metrics to gauge which youths use Project YES and whether they view Project YES as valuable, helpful, and user-friendly.

## Methods

### Recruitment

The participants in this project were youths aged 11 through 17 years from San Antonio, Texas, and the surrounding areas (within a 100-mile [161 km] radius) who interacted with the Project YES platform between April 31, 2021, and December 31, 2021. The participants learned about Project YES through several sources, including paid advertisements on Instagram (Meta Platforms, Inc), friends, teachers, and the University of Texas Teen Health (UTTH) team. UTTH provided 12 opportunities for representatives from potential community partners (eg, schools and community centers) and community members to receive an orientation to Project YES, which covered the following topics: (1) overview of Project YES, (2) introduction to Project YES in San Antonio, (3) goals for Project YES San Antonio, (4) what youths will do in Project YES, (5) community partner roles and responsibilities, and (6) additional resources. The purpose of the orientation was to provide the community with an understanding of Project YES and resources to refer youths to the program. A total of 79 people attended the orientation. From April 1, 2021, to June 30, 2021, of those who attended the orientation, 2 (3%) school partners and 4 (5%) community organizations reported referring youths to Project YES. The partners made 2763 referrals during this reporting period.

To facilitate youth-level recruitment efforts, UTTH created advertising postcards with the QR code to access the website, along with phone numbers, on the back of the postcard for youths who may be in crisis and need immediate assistance. These postcards were distributed to youths in schools, community organizations, counseling offices, and clinics and anyone interested in the program. During the project year, a total of 12,839 postcards were distributed. UTTH also created flyers, with the assistance of the Stony Brook University team, which were posted on the Lab for Scalable Health’s Instagram page. These were also posted on UTTH’s social media sites and the UTTH program website and added to UTTH staff’s email signature blocks.

To facilitate community partners’ efforts to increase Project YES’s uptake, UTTH created a social media toolkit to aid partners in sharing Project YES with the youths in their organization. This comprehensive guide has information on the need for the project, the evidence base, information on how to engage with Project YES, sample social media posts, a sample script for introducing Project YES to young people, and frequently asked questions. When possible, UTTH also attended health fairs or school functions to inform attendees about Project YES, with an express focus on the San Antonio area.

### Facilitating the Uptake of Project YES in Schools

Owing to COVID-19–related public health restrictions at the beginning of the 2021 school year, visitors were not allowed on campus for Project YES–related presentations. However, in October 2021, UTTH received the opportunity to present Project YES during a physical education class to middle school– and high school–aged students at a local school district. UTTH coordinated with district-based physical education coaches to offer Project YES to the students on the campus. Each coach reserved the computer laboratory or gymnasium as per availability. The coaches asked the students to bring their school laptops to the session. During these sessions, the students were informed about the program and logged in using the QR code for their phone or a computer desktop or laptop. There were some challenges with poor internet connectivity, specifically in the gymnasium, which prevented some students from completing an SSI within Project YES. Subsequently, this process was replicated with a second school district partnering with UTTH, again targeting the district’s middle school– and high school–aged students.

### Ethical Considerations

Before launching Project YES, all procedures were reviewed and deemed “exempt” (as a program evaluation) by the UT Health San Antonio’s institutional review board (IRB).

### Project Procedures

#### Overview

There are no inclusion or exclusion criteria for Project YES, as it is publicly accessible, and the data collected are nonidentifiable. Although the Project YES website states that the activities are designed for youths aged >18 years (primarily preadolescent and adolescent youths), individuals of any age may choose to participate. The participants are required to report their age range (11 to 13, 14 to 16, or 17 years) and whether they are aged ≥18 years (yes or no) before initiating Project YES. The participants are required to report whether they were from San Antonio (or within 100 miles; yes or no). Using these data, we limited the analyses to our target population (youths aged 11 to 17 years from San Antonio).

The structure of Project YES has been described in a previous manuscript [10]. To summarize, Project YES is a nonrandomized, ongoing, and exploratory program evaluation whose original procedures were preregistered on Open Science Framework [34]. Parent permission is not required to participate in Project YES (waived by the University’s IRB) to minimize access barriers, including discomfort disclosing psychological distress (as many parents are not aware of their children’s depressive symptoms), and to ensure that youths remain unidentifiable.

Once the participants begin Project YES, advance past the “Project Information” page, and agree to participate, they are prompted to provide nonidentifying demographic information. Next, the participants select 1 of 3 SSIs to complete before completing pre-SSI questionnaires, the SSI itself, and post-SSI questionnaires, which were designed to measure the SSI’s short-term effects and acceptability. Finally, the participants have the option to anonymously share their “best advice for others dealing with depression, anxiety, or stress,” which is posted on the “YES Advice Center” for others to read.

The current version of Project YES was adapted in several ways to better serve the San Antonio community. Specifically, Project YES was translated entirely into Spanish, including the pre- and postintervention questionnaires and the 3 digital SSIs. Before participating in the SSIs, the participants are given the option to select the language (either English or Spanish) in which they would like to hear or view the SSI. In addition, the question “Do you live in San Antonio, TX, or the surrounding areas (ie, within 100 miles, like Atascosa, Medina, Bandera)?” was added. Using these data, we limited the analyses to our target population (youths from San Antonio or its surrounding areas).

#### Inviting Teens to Share Their Stories for Inclusion in Project YES

San Antonio youth volunteers were invited to contribute their lived experience–based stories of coping with depression, anxiety, and related difficulties for inclusion in the culturally adapted Project YES SSIs. The procedures for youths’ involvement in SSI adaptation were reviewed and deemed “Not Regulated Research” by the University IRB. Participating youths were members of the UTTH’s Youth Leadership Council who expressed interest in sharing their stories and being a part of a program geared toward helping San Antonio–based teens. UTTH also shared the opportunity with teenagers via their Facebook (Meta Platforms, Inc) and Instagram pages. Youths interested in sharing their stories were then contacted and provided with further information about Project YES and the process of gathering stories. In total, 14 San Antonio youths (aged 12 to 17 years) elected to contribute their personal stories of mental health and coping to Project YES; parents’ or guardians’ written permission was obtained for all 14 volunteers. Ultimately, UTTH gathered 33 stories from these 14 youths, all of which were integrated into Project YES.

#### Collecting Youth Stories for Inclusion in Project YES

UTTH and Stony Brook University representatives met with the youth volunteers via Zoom (Zoom Video Communications, Inc), in groups of 3 to 5, on multiple dates to ensure that each volunteer had an opportunity to write and submit their stories to UTTH. Each volunteer was asked to attend 2-hour-long Zoom meetings to construct their stories for inclusion in the interventions. Our focus on adapting and embedding San Antonio youths’ personal stories into Project YES reflects Heim and Kohrt’s Cultural Adaptation Framework for Scalable Interventions [32], which highlights the core importance of integrating cultural concepts of distress into adapted interventions. In other words, we aimed to situate each SSI’s core concepts and skills (eg, behavioral activation and self-kindness) within San Antonio youths’ lived experiences, perspectives, and narratives of *how they have experienced and understood their own distress* as well as *how a particular skill has helped them overcome or manage that experience of distress.* By framing the purpose and relevance of the SSI content through the lens of San Antonio youths’ voices and perspectives and by making key “non-specific” adaptation to signal cultural relevance (eg, a full Spanish translation), we aimed to increase the program’s relevance to users’ worldviews, thus improving the acceptability and clinical impact of the interventions.

Stony Brook University and UTTH staff facilitated small breakout sessions. Confidentiality was reviewed at the beginning of the session, and a mental health professional was on hand to provide assistance if any youth volunteer needed help when telling their stories. The youths were asked questions about their experiences at school, home, and community where they may have been stressed, anxious, or challenged and how they overcame those situations. The youth volunteers verbally shared their answers, and one of the facilitators scribed each volunteer’s stories. The youths were also offered the option of writing their stories, rather than narrating them, based on their comfort level and personal preference. At the end of the meeting, a written summary of their stories was provided to each youth volunteer; they were then invited to provide feedback and edit their transcribed stories to ensure that each story optimally reflected their lived experience and perspective. All youths’ stories were included (anonymously or using pseudonyms) in the final, adapted version of Project YES for San Antonio youths—either within one of the digital SSIs or immediately after the SSIs as part of a collection of “coping narratives” from peers, which all Project YES users had the chance to review.

#### Translating Project YES Into Spanish

UTTH staff, who were either native Spanish speakers or fluent in Spanish, translated each story into Spanish. Several youth volunteers were bilingual and translated their own stories into Spanish. The Project YES home page [35] and all 3 SSIs within Project YES were also translated into Spanish by an externally hired professional translator, along with input from UTTH.

#### Recording Youths’ Stories for Inclusion in Project YES

Within the Project YES SSIs, narratives from young people are available as both *written stories* and *audio recordings* to help users feel more connected to their peers’ mental health and coping narratives. Therefore, UTTH contracted the UT Health San Antonio Media Services Department to provide the studio and equipment to record all stories in English and Spanish. All youth volunteers were offered the opportunity to self-record their own stories, although none was required to do so.

Multiple recording dates were arranged to accommodate the youth volunteers’ school schedules. Each volunteer received a copy of the story or stories they had written, and they were guided by the media services director on how to properly use the recording equipment. The youths were able to make changes to their stories’ wording if deemed necessary, and the director lent periodic guidance to improve the flow and intonation. Two of the bilingual youth volunteers recorded each of their stories in Spanish. Media services staff edited the files to accommodate the multiple takes in each volunteer’s recording and then provided a final file to UTTH (1 audio file per story). After the recordings, the youth volunteers were provided with a “swag bag” and gift cards in appreciation for their time, dedication, and contribution to the project (US $85 for each volunteer, for several hours of effort). Parents of the youth volunteers also received gift cards for supporting their children in this work (US $50).

### Interventions

#### Overview

The SSIs in Project YES were designed to incorporate 4 design features common across effective, self-guided SSIs for youth mental health, as proposed by Schleider et al [10]. Each SSI within Project YES (1) incorporates true stories from trustworthy individuals, including older peers (in this case, stories and narratives from San Antonio youths; refer to Multimedia Appendix 1 for examples) and scientific experts; (2) use brain science–based explanations to increase the credibility of the content; (3) empowers users to act as an “expert” or “helper” throughout the SSI by leveraging their own experience to offer advice to others; and (4) offers guided writing activities, often referred to as “saying-is-believing” or “self-persuasion” exercises. A detailed explanation of these 4 SSI design features has been provided elsewhere [10]. The culturally adapted version of Project YES, which was included in the present evaluation, incorporated new narratives constructed with San Antonio youths via the processes described earlier.

Materials for all SSIs within Project YES are publicly available via the Open Science Framework (Project Personality [36], Project CARE [37], and The ABC Project [38]). Each SSI is a 30-minute, self-delivered program. All the materials that youths who participate in Project YES view during the program are publicly viewable on the Project YES website [35].

There are currently 2 published reports on the preliminary acceptability and utility of the Project YES interventions across all youths who completed an intervention between September 2019 and August 2020 [10,12]. The results demonstrated acceptability and positive effects on both hopelessness and perceived agency for all 3 interventions [10].

#### Project Personality

The original (unadapted) version of Project Personality has been described elsewhere [10], and its structure is summarized here for convenience. Project Personality opens with an introduction to the human brain and a lesson on neuroplasticity. This SSI includes true stories from older youths, which explain their views that personality traits are malleable and describe moments in their lives when they used “growth mindsets” to persevere during setbacks. Project Personality also explains how and why one’s personality can change and includes an exercise in which youths incorporate scientific information in the notes they write to younger youths about people’s capacity for change. This SSI was adapted to include stories provided by San Antonio youths for San Antonio youths. Project Personality has shown effectiveness in multiple trials [32,39,40].

#### Project CARE

The original (unadapted) version of Project CARE has been described elsewhere [10]; however, its structure is summarized here for convenience. Project CARE begins with an introduction to the science that explains why many youths believe that they must dislike themselves to be successful and thus fear self-compassion. This SSI contains scientific evidence and true stories from other youths explaining that being self-compassionate predicts greater social and academic success. Evidence-based tips for overcoming the fear of self-compassion–based obstacles are provided. Youths have the opportunity to author notes to younger adolescents using scientific information to explain the different ways in which self-kindness can be beneficial. This SSI was adapted to include stories provided by San Antonio youths for San Antonio youths.

#### The ABC Project

The original (unadapted) version of The ABC Project has been described elsewhere [10]; however, its structure is summarized here for convenience. The ABC Project incorporates components of behavioral activation and introduces the idea that participating in value-based activities can combat low self-esteem and a sad mood. Through The ABC Project, youths receive psychoeducation about depression, specifically about how one’s behavior can shape one’s thoughts and feelings. Youths are walked through a life-values assessment in which they identify key areas from which they gain enjoyment and meaning; next, they identify and personalize 3 activities to focus on for change. Finally, The ABC Project offers an activity in which youths write about engaging in each of their targeted activities and the benefits that may come from engaging in them; they also pinpoint obstacles that may stop them from doing the activities and outline strategies to overcome the obstacles.

### Measures

The following measures on Project YES are the same as those that prior reports have described; however, in the context of this data collection, we focused on the version of Project YES that contains both the English and Spanish versions.

#### Demographics

The participants identified their age range (age was provided in ranges to maintain anonymity: 11 to 13, 14 to 16, or 17 years); biological sex; gender identity; sexual orientation; race and ethnicity; how they learned about Project YES; and whether they were from San Antonio, Texas, or the surrounding areas. If they selected “Yes,” they were asked in what part of San Antonio they lived (north, east, south, or west).

#### Mood and Feelings Questionnaire–Short

The Mood and Feelings Questionnaire—Short (SMFQ) is a valid, reliable, and commonly used assessment for depression symptoms in youths, which was previously translated into English and Spanish [41]. Before using an SSI, the participants rated their agreement with 13 statements reflecting thoughts and feelings over the previous 2 weeks (eg, “I felt lonely,” “I felt miserable or unhappy,” and “I felt I was no good anymore”) on a 3-point Likert scale (0=“not true,” 1=“sometimes,” and 2=“true”). Internal consistencies among the San Antonio youths who completed Project YES in English and Spanish, respectively, were Cronbach α=.93 and Cronbach α=.91. Note that the SMFQ does not measure suicidal ideation or suicidality, which are not assessed in the context of Project YES.

#### State Hope Scale

The State Hope Scale is a 6-item self-report scale created to evaluate hope in youths. The State Hope Scale includes 2 reliable subscales: agency and pathways [42]. The “agency” subscale measures one’s perceived ability to create plans and work to achieve goals (eg, “I can think of many ways to reach my current goals”); the “pathways” subscale reports one’s perceived success in meeting one’s goals (eg, “At this time, I am meeting the goals I have set for myself”). For Project YES, we anticipated shifts in *agency* but not in *pathways* scores. This is because participants may not have opportunities to pursue their goals in new ways immediately after SSI. Thus, hope was indexed using the 3-item agency subscale of the State Hope Scale. The participants rated 3 statements immediately before and after intervention to reflect how they felt about themselves at that moment on an 8-point Likert scale (1=“definitely false” to 8=“definitely true”). Internal consistency among the San Antonio youths who completed Project YES in English was Cronbach α=.78 and Cronbach α=.91 before and after SSI, respectively. Internal consistency among the San Antonio youths who completed Project YES in Spanish was Cronbach α=.86 and Cronbach α=.86 before and after SSI, respectively.

#### Beck Hopelessness Scale-4

The Beck Hopelessness Scale-4 is a reliable, commonly used, shortened version of the 20-item scale that measures hopelessness in youths [42,43]. The participants rated 4 statements immediately before and after intervention to indicate their sense of hopelessness “right now, in this moment” on a 4-point Likert scale (0=“absolutely disagree,” 1=“somewhat disagree,” 2=“somewhat agree,” and 3=“absolutely agree”). Internal consistency among the San Antonio youths who completed Project YES in English was Cronbach α=.87 and Cronbach α=.91 before and after SSI, respectively. Internal consistency among the San Antonio youths who completed Project YES in Spanish was Cronbach α=.76 and Cronbach α=.91 at before and after SSI, respectively.

#### Self-hate Scale

The Self-hate Scale is a reliable, 3-item measure designed to assess feelings of self-hate ([15,44]; adapted from the study by Turnell et al [44]). Immediately before and after the intervention, the participants rated how true each of 3 statements was for them at that moment (“I hate myself,” “I feel disgusted when I think about myself,” and “I feel ashamed of myself”) on a 6-point Likert scale (1=“not at all true for me” to 6=“very true for me”). Internal consistency among the San Antonio youths who completed Project YES in English was Cronbach α=.93 and Cronbach α=.96 before and after SSI, respectively. Internal consistency among the San Antonio youths who completed Project YES in Spanish was Cronbach α=.86 and Cronbach α=.95 at before and after SSI, respectively.

#### Perceived Change in Hopelessness and Problem-solving

The participants were asked 2 questions immediately after intervention, which evaluated their perceived change in hopelessness and ability to solve problems. Questions were developed for this study based on established guidelines for assessing subjectively perceived change following an intervention [45]. These questions asked, “to what extent are you feeling hopeless right now?” and “to what extent are you able to solve the problems facing you right now?” when “compared to before doing this activity.” Both perceived change in hopelessness and problem-solving ability were rated on a 5-point Likert scale (“much more hopeless” to “a lot less hopeless”; “much less able to solve problems” to “a lot more able to solve problems”). These measures were developed based on previously established methods used to calculate the “smallest effect size of interest” (the smallest possible effect size associated with a detectable, subjective change within individuals) [46].

#### Program Feedback Scale

The Program Feedback Scale (PFS) is commonly used to evaluate the acceptability and user perceptions of SSIs [16,20,46,47]. It asks participants to rate their level of agreement with 7 statements that indicate the perceived acceptability and feasibility of the SSI they chose (eg, “I enjoyed the program”) on a 5-point Likert scale (1=“really disagree,” 2=“disagree,” 3=“neutral,” 4=“agree,” and 5=“totally agree”). The PFS was adapted from the existing, validated acceptability assessments of digital interventions. To exclude items that did not apply to web-based SSIs (ie, items that reference one’s interest in revisiting the program), adaptations from the existing scales were necessary. The PFS also assesses open-response feedback from participants. The PFS item scores can be evaluated either individually or across items via a mean score. Internal consistency across PFS items for the youths who completed Project YES in English and Spanish was Cronbach α=.93 and Cronbach α=.87, respectively; mean responses to *each* PFS item were evaluated separately to gain insight into the acceptability in specific domains (eg, ease of use and understanding and enjoyability).

### Analytic Plan

#### Sample Characterization and Use Patterns

To evaluate the use patterns of Project YES, we quantified the number of youths who started Project YES and those who chose, began, and completed an SSI. In addition, we identified which SSI the youths chose as well as the demographics, symptom levels, hopelessness, and self-hate levels for youths who chose, began, and completed an SSI. Responses from Spanish and English respondents were evaluated separately. We calculated the overall and item-specific means for youths who completed an SSI and the PFS to evaluate each SSI’s feasibility and acceptability. Mean scores of >3 for any PFS item demonstrated item endorsement (eg, satisfactory acceptability). Mean scores of >3 across all items demonstrated overall SSI acceptability. Descriptive statistics for pre-to-post SSI “perceived change” items were calculated using responses from the subsample of individuals who completed an SSI. Mean ratings >0 for each item demonstrated a subjectively detectable overall pre-to-post SSI change for that dimension (problem-solving ability or hopelessness).

#### SSI Effects on Proximal Outcomes

We calculated within-group effect sizes (Cohen *d*, including 95% CIs), which reflected the change in pre-to-post SSI levels of each post-SSI outcome variable (agency, hopelessness, and self-hate) across SSIs. Because the previous reports of outcomes for Project YES did not indicate significant differences across SSI selection [10,12], all outcome data were collapsed for analytical purposes. There are several ways to compute Cohen *d* for within-subject designs [48]. Here, we report Cohen *d*_z_, which accounts for within-subject correlations between pre- and post-SSI measures:


Cohen *d_z_* = M_diff_ / √(∑((*x*_diff_ − *M*_diff_)^2^ / (*N* − 1)) **(1)**

#### Data Quality and Exclusions

Within self-report surveys, particularly within community-based program evaluations (such as this project), it is common for a subset of participants not to pay sufficient attention or put sufficient effort into providing valid questionnaire responses. Because prior work demonstrates that even a small percentage of invalid responses can undermine the interpretability of results [49], it is necessary to identify and filter careless or invalid effort (CIE) responses to optimize the interpretation and improve the accuracy of results. Because of the naturalistic nature of data collection in this project, we used a filtering strategy that prioritized specificity (detecting and removing *only the most clearly CIE* responses) over sensitivity (detecting and removing *all potentially CIE responses*). Specifically, rather than filtering data based on less-diagnostic metrics (such as time spent on the survey), we identified and excluded responses that demonstrated a “straightlining” pattern, whereby an individual provides identical responses to all survey items regardless of their content and direction [50]. Of the various approaches to identifying CIE responses, straightlining has been shown to have the most pronounced impact on data properties [50]. Here, we defined “straightlining” as providing numerically identical responses across *all 3* Likert-scale based outcomes, either before SSI or after SSI (the Beck Hopelessness Scale-4 item version, for which higher numeric responses reflect poorer functioning; the State Hope Scale—Agency subscale, on which higher numeric responses reflect more positive functioning; and the Self-hate Scale, on which higher numeric responses reflect poorer functioning). To optimize the validity of the results, the individuals who followed “straightlining” response patterns (ie, responding to all pre- or post-SSI Likert-scale items with “1,” regardless of item direction or content) were removed from the effect size computation analyses.

All available data meeting the above-described inclusion criteria were used for each test described above, and data from Spanish and English respondents were evaluated separately. As use patterns (including attrition) were of direct empirical interest, missing data rates are reported but not imputed. Anonymized data and codes for all analyses are available via the Open Science Framework.

## Results

### Sample and Use Patterns in Project YES

Between April and December 2021, a total of 1801 San Antonio youths began Project YES, of whom 894 (49.64%) completed a 30-minute, SSI. Specifically, 1705 youths began Project YES in English and 855 of them completed it (completion rate: 50.15%). A total of 96 youths began Project YES in Spanish and 39 of them completed it (completion rate: 41%). The overall completion rate of 49.64% is considerably higher than those previously observed for Project YES (34.2%). The youths who participated in Project YES were demographically diverse. Across all youths who began Project YES in English, 94.78% (1616/1705) identified as people of color, 53.96% (920/1705) as biologically female, and 39.3% (670/1705) as nonheterosexual. There were 1464 youths who began an SSI activity in English. Among those who started an SSI in English, 29.71% (435/1464) chose Project CARE (257/435, 59.1% completion rate among those who began the SSI), 23.43% (343/1705) chose The ABC Project (206/343, 60.1% completion rate among those who began the SSI), and 46.86% (686/1464) chose Project Personality (392/686, 57.1% completion rate among those who began the SSI). Completion rates did not substantially differ between the 3 SSIs.

Across all youths who began Project YES in Spanish, 99% (95/96) identified as people of color, 43% (41/96) as biologically female, and 33% (32/96) as nonheterosexual. There were 77 youths who began an SSI in Spanish. (Notably, beginning an SSI requires the completion of all pre-SSI questionnaires; as such, 96 youths began Project YES in Spanish, but only 77 youths completed the pre-SSI questionnaires and initiated an intervention in Spanish.) Among those who started an SSI in Spanish, 29% (22/77) chose Project CARE (8/22, 36% completion rate among those who began the SSI), 25% (19/77) chose The ABC Project (11/19, 58% completion rate among those who began the SSI), and 47% (36/77) chose Project Personality (20/36, 56% completion rate among those who began the SSI). Completion rates did not substantially differ between the 3 SSIs. Across the total sample (both Spanish and English completers), sexual orientation (nonheterosexual vs heterosexual) was associated with the odds of completion; specifically, nonheterosexual participants were less likely to complete their selected SSI. Across the total sample, racial and ethnic identity, biological sex (female vs intersex vs male), and depressive symptom severity (total SMFQ score) were not associated with the odds of completion.

Youths who completed Project YES in English in its entirety spent 34.6 (SD 21.2; median 32.7; range 6.27-409) minutes on the YES website on average; this included the time spent on all questionnaires, the SSI they chose, and authoring anonymous advice for the YES Advice Center. Youths who completed Project YES in Spanish in its entirety spent 40.3 (SD 20.0; median 37.2; range 19.1-105) minutes on the YES website on average. Across all youths who accessed the YES website (including those who neither started nor completed an SSI), the average amount of time spent on Project YES was 29.8 minutes for the English version and 28.6 minutes for the Spanish version.

### Did the Youths Perceive Project YES as Acceptable?

The youths who completed both an SSI and the PFS (794/1801, 44.09%) in English, collapsing both across and within each SSI, found Project YES to be acceptable. Overall, these youths rated the SSI they chose as enjoyable (3.57/5.00), easy to understand (3.94/5.00), easy to use (3.99/5.00), likely to help their peers (3.89/5.00), and worth recommending to others (3.77/5.00). In addition, the youths endorsed that they tried their hardest on the SSI they chose (3.65/5.00) and that they agreed with the SSIs message (4.02/5.00).

The youths who completed both an SSI and the PFS (37/1801, 2.05%) in Spanish, collapsing both across and within each SSI, found Project YES to be acceptable. Overall, these youths rated the SSI they chose as enjoyable (3.95/5.00), easy to understand (4.05/5.00), easy to use (4.22/5.00), likely to help their peers (4.14/5.00), and worth recommending to others (4.24/5.00). In addition, the youths endorsed that they tried their hardest on the SSI they chose (4.16/5.00) and that they agreed with the SSIs message (4.27/5.00; Table 1).

**Table 1:** Means and SDs of the Program Feedback Scale items among single-session intervention (SSI) completers, across all SSIs in English and Spanish.

Item	All SSIs—English, mean (SD)	All SSIs—Spanish, mean (SD)
Enjoy	3.57 (1.04)	3.95 (1.13)
Understood	3.94 (0.97)	4.05 (1.15)
Easy to use	3.99 (1.00)	4.22 (1.03)
Tried hardest	3.65 (1.10)	4.16 (1.14)
Helpful	3.89 (1.11)	4.14 (1.23)
Recommend to friend	3.77 (1.17)	4.24 (0.93)
Agree with message	4.02 (1.03)	4.27 (1.04)
Full scale	3.84 (0.88)	4.15 (0.83)

The respondents who completed the PFS in English left the following comments in it: “User friendly and the examples of kids my age made me feel understood and not alone”; “I like that it is interactive and has reflection built in. The balance of science and real life experiences was great”; “I learned how to be nicer to myself”; and “It was very helpful for me to improve on things.” The respondents who completed the PFS in Spanish left the following comments: “Me gusto que le cambió la vida a otros y ami misma” (I liked that it changed the lives of others and myself); “Que puede aplicar mis futuros problemas en el ejemplo” (That I can apply my future problems in the example); and “Me gusto como me hizo pensar en vez de nada más dando ejemplos sobre otros” (I liked how it made me think instead of just giving examples about others).

### Did Hopelessness, Self-hate, and Perceived Agency Improve From Before to After Project YES?

To optimize the validity and accuracy of the effect size estimates, we identified and excluded the youths who demonstrated a “straightlining” pattern of responses across all pre- or post-SSI Likert-scale items that assessed the psychosocial outcomes of interest (hopelessness, self-hate, and agency). In total, 3.9% (33/855) and 2.9% (25/855) of the English-language respondents showed a straightlining pattern in the pre-SSI and post-SSI surveys, respectively; 3% (1/39) and 8% (3/39) of the Spanish-language respondents showed a straightlining pattern in the pre-SSI and post-SSI surveys, respectively. The results reported subsequently reflect the effect sizes observed after the removal of these responses.

SSI short-term utility was tested both across SSIs, as they have shared principles and common structures, and separately, as each SSI is distinct in content. The effect sizes Cohen *d*_z_ and 95% CIs both across SSIs and for each SSI are reported (Table 2). Across all 3 SSIs in the English version of Project YES, the youths reported significant improvements in all proximal outcomes pre-to-post SSI. Regarding overall reductions in hopelessness, small-medium effects emerged (2-tailed *t*_712_=8.87; *P*<.001; 95% CI 0.26-0.41, Cohen *d*_z_=0.33), with post-SSI hopelessness showing a 57.6% chance of being lower than pre-SSI hopelessness (per the “common language effect size” estimate; refer to the study by Lakens [48]). For overall reductions in self-hate, small-medium effects emerged (*t*_698_=7.11; *P*<.001; 95% CI 0.19-0.34, Cohen *d*_z_=0.27), with post-SSI self-hate showing a 55% chance of being lower than pre-SSI self-hate. For overall improvements in perceived agency, small-medium effects emerged (*t*_707_=6.54; *P*<.001; 95% CI 0.17-0.32, Cohen *d*_z_=0.25), with post-SSI perceived agency showing a 56.2% chance of being higher than pre-SSI perceived agency.

Across all 3 SSIs in the Spanish version of Project YES, the youths reported a significant improvement in self-hate from before to after SSI. For overall reductions in self-hate, small-medium effects emerged (*t*_29_=2.05; *P*=.049; 95% CI 0.001-0.74, Cohen *d*_z_=0.37), with post-SSI self-hate showing a 55% chance of being lower than pre-SSI self-hate (per the “common language effect size” estimate; refer to the study by Lakens [48]). The youths did not report a substantial improvement in hopelessness or perceived agency.

SSI-specific effect sizes were not calculated, as the programs do not appear to have differential effects; therefore, there was no reason to expect differential effects in this study [10,15].

**Table 2:** Means, SDs, and effect sizes by single-session intervention (SSI) and across all SSIs in English and Spanish^a^.

Outcome variable			English									Spanish						
			ABC Project		Project CARE		Project Personality		All SSIs		ABC Project			Project CARE		Project Personality		All SSIs
**Agency**																		
	Before SSI, mean (SD)	5.3 (1.7)		5.2 (1.8)		5.5 (1.7)		5.3 (1.7)		6.3 (1.5)			5.9 (1.6)		5.6 (2.0)		5.8 (1.8)	
	After SSI, mean (SD)	5.8 (1.9)		5.5 (2.1)		5.9 (1.9)		5.7 (2.0)		6.6 (1.8)			5.1 (2.7)		5.8 (2.0)		5.9 (2.1)	
	Cohen *d*_z_ (95% CI)	*0.25 (0.10 to 0.40)* ^b^		*0.24 (0.10 to 0.38)*		*0.25 (0.14 to 0.36)*		*0.25 (0.17 to 0.32)*		0.07 (−0.56 to 0.69)			0.39 (−0.46 to 1.20)		*0.57 (0.01 to 1.11)*		0.12 (−0.23 to 0.47)	
**Hopelessness**																		
	Before SSI, mean (SD)	1.2 (0.9)		1.1 (0.9)		1.0 (0.8)		1.0 (0.9)		1.0 (0.8)			1.2 (1.0)		1.9 (0.8)		1.0 (0.8)	
	After SSI, mean (SD)	0.8 (0.9)		0.8 (0.8)		0.7 (0.8)		0.8 (0.9)		1.1 (1.0)			1.1 (1.2)		1.7 (0.9)		0.9 (1.0)	
	Cohen *d*_z_ (95% CI)	*0.37 (0.22 to 0.52)*		*0.38 (0.24 to 0.52)*		*0.28 (0.17 to 0.39)*		*0.33 (0.26 to 0.41)*		0.34 (−0.31 to 0.97)			0.17 (−0.58 to 0.91)		0.02 (−0.46 to 0.49)		0.00 (−0.34 to 0.34)	
**Self-hate**																		
	Before SSI, mean (SD)	2.7 (1.8)		2.6 (1.7)		2.3 (1.6)		2.5 (1.7)		1.9 (1.3)			2.1 (1.6)		2.2 (1.6)		2.1 (1.5)	
	After SSI, mean (SD)	2.2 (1.6)		2.3 (1.6)		2.0 (1.5)		2.1 (1.6)		2.5 (2.0)			1.9 (2.0)		1.4 (0.8)		1.8 (1.6)	
	Cohen *d*_z_ (95% CI)	*0.37 (0.22 to 0.53)*		*0.21 (0.07 to 0.35)*		*0.25 (0.14 to 0.36)*		*0.27 (0.19 to 0.34)*		0.10 (−0.56 to 0.75)			0.55 (−0.34 to 1.39)		0.47 (−0.07 to 0.99)		*0.37 (0.00 to 0.74)*	

^a^For hopelessness and self-hate measures, lower scores indicate better functioning, and for perceived agency, higher scores indicate better functioning. Where applicable, Cohen *d* values are corrected (multiplied by −1.0) such that positive values indicate greater improvements from before to after SSI.

^b^Significant improvements are italicized.

### Did the Youths Subjectively Detect Changes in Hopelessness and Problem-Solving Ability From Before to After Project YES?

Among the youths who completed Project YES in English, after the intervention, 32.9% (259/785) reported feeling “much less hopeless,” 30.8% (242/785) felt “a little less hopeless,” 23.5% (185/785) felt “the same amount hopeless,” 5.3% (42/785) felt “a little more hopeless,” and 7.5% (59/785) felt “a lot more hopeless” compared with before beginning the SSI. Separately, 27.2% (214/785) of the youths reported feeling “much more able to solve problems,” 31.3% (246/785) felt “a little more able to solve problems,” 28.6% (225/785) felt “the same amount able to solve problems,” 7% (55/785) felt “a little less able to solve problems,” and 5.9% (47/785) felt “a lot less able to solve problems” compared with before beginning the SSI.

Among the youths who completed Project YES in Spanish, after the intervention, 38% (14/37) reported feeling “Mucho menos desesperanzado” (“much less hopeless”), 27% (10/37) felt “Un poco menos desesperanzado” (“a little less hopeless”), 8% (3/37) felt “Lo mismo de desesperanzado” (“the same amount hopeless”), 8% (3/37) felt “Un poco más desesperanzado” (“a little more hopeless”), and 19% (7/37) felt “Mucho más desesperanzado” (“a lot more hopeless”) compared with before beginning the SSI. Separately, 43% (16/37) of the youths reported feeling “Mucho más capaz de resolver los problemas” (“much more able to solve problems”), 24% (9/37) felt “Un poco más capaz de resolver los problemas” (“a little more able to solve problems”), 22% (8/37) felt “La misma capacidad de resolver los problemas” (“the same amount able to solve problems”), 8% (3/37) felt “Un poco menos capaz de resolver los problemas” (“a little less able to solve problems”), and 3% (1/37) felt “Mucho menos capaz de resolver los problemas” (“a lot less able to solve problems”) compared with before beginning the SSI.

## Discussion

### Principal Findings

This academic-community partnership project sought to evaluate the acceptability and short-term utility of an open-access, culturally adapted digital platform for English- and Spanish-speaking adolescents, providing three 30-minute self-administered SSIs to young people living in the city of San Antonio, Texas. From before to after the intervention, the youths who completed Project YES in English reported short-term improvements in hopelessness (Cohen *d*=0.33), self-hate (Cohen *d*=0.27), and perceived agency (Cohen *d*=0.25). In addition, the youths who completed Project YES in Spanish reported improvements in self-hate (Cohen *d*=0.37) from before to after the intervention. The youths who completed an SSI in either language rated it as enjoyable, easy to understand, likely to help peers, and worth recommending to others. On the basis of the largely overlapping CIs for Cohen *d*_z_, no evidence emerged for the differential effects of individual SSIs based on proximal outcomes. That is, the youths who completed Project YES reported similar short-term improvements in self-hate, agency, and hopelessness regardless of the specific SSI they opted to complete—consistent with prior trials comparing the impacts of the SSIs in Project YES [10,15].

Findings build on a growing body of evidence for single-session digital interventions as scalable and accessible mental health tools for young people [10,15]. Past studies have reported the acceptability and utility of culturally adapted internet-based interventions for mental disorders [51]. This project provides evidence of the acceptability of cultural adaptations of SSIs. The youth stories, focus groups, and translations involved teens directly from the local community during the adaptation process. Furthermore, the mean SMFQ score for the sample was 9.97, with users’ scores ranging from 0 to 26, suggesting usefulness for both young people with lower symptoms and those with higher symptoms (ie, as a preventive tool), and for youths already experiencing substantial distress. This project suggests its utility and acceptability as a mental health resource when adapted for the needs of a specific underserved community or population of San Antonio, Texas.

The composition of the sample was highly representative of the community and very diverse relative to most youth mental health studies, with >94% the teens identifying as people of color (predominantly Latino, Latina, Latine, or Hispanic; African American; Asian; and other). Therefore, the findings of this study are likely to be generalizable to other youths in this area. Furthermore, the high percentage of lesbian, gay, bisexual, transgender, queer and more (LGBTQ+) youths in the sample (approximately 33%-39%) provides evidence for the project’s generalizability and usability. However, LGBTQ+ youths were less likely to complete their selected SSI, and further work should investigate this notable finding. The sample in this study consisted of a more even gender or biological sex balance than those in other SSI trials [10,11,15], potentially because of community-facilitated intervention dissemination (eg, in classrooms). Future work on digital mental health supports may benefit from using community-facilitated dissemination as a tool for engaging more males.

Similar to other web-based SSI evaluations [10,15,52], the youths in this project showed an SSI completion rate of 49.6%. Across interventions, among the youths who began an SSI, 36.6% to 60.1% completed the full activity. This relatively high completion rate provides further evidence of youth acceptability for web-based SSIs, as prior studies of self-guided digital mental health programs have shown substantially lower completion rates (0%-28%, on average) [7]. Furthermore, pre-SSI youth dropout (ie, dropout occurring during the pre-SSI questionnaire stage) was only 14.4% in this project, which was substantially lower than the pre-SSI dropout rate observed in a previous program evaluation of Project YES (37.2%) [17]. Proactive, community-partnered dissemination efforts (rather than relying exclusively on social media–based dissemination) may have contributed to the notably higher completion rates in this project. Cultural adaptations may have likewise increased the youths’ engagement with the SSIs and willingness to complete the programs in full.

In future efforts to disseminate youth-focused web-based SSIs, to further minimize pre-SSI dropouts (which was 14.4% in this project), we strongly suggest minimizing or eliminating pre-SSI questionnaires. As evidence for the acceptability, utility, and safety of these SSIs in diverse populations continues to grow, offering SSIs beyond the context of program evaluations and randomized trials will become increasingly feasible. Relatively few youths chose to complete the SSIs in Spanish. Among the youths who did complete Project YES in Spanish, the completion rates did not differ as a function of the selected SSI, and they reported all SSIs as acceptable and likely to help. Many youths in the San Antonio area are bilingual English-Spanish speakers. Although many of their parents might have been monolingual Spanish speakers, the youths may have chosen to complete Project YES in English out of personal preference or comfort level. Nonetheless, it is possible that the mere availability of a Spanish-translated version of Project YES might have been viewed positively by San Antonio youths. For example, it might have signaled that the program was built with their needs and community in mind, thereby strengthening the feelings of belonging and relatedness in the context of the SSIs. Future studies are needed to empirically examine this possibility. However, it is notable that the exact percentages of primarily Spanish- and English-speaking students at the participating schools were not publicly available; therefore, the possibility of differential Project YES engagement based on primary language remains unclear. Additional research is needed to gauge whether treatment access disparities are maintained or reduced by making Spanish-language youth mental health supports freely available.

Although our results demonstrated overall acceptance of the culturally adapted English and Spanish SSIs in Project YES, our project is not without limitations. As this was a nonexperimental, observational, and anonymous project because of the goals of the study, follow-up data were not collected; therefore, the findings should be interpreted cautiously. However, the same SSIs (albeit not the present culturally adapted versions) have demonstrated efficacy in large randomized control trials across 3- to 9-month follow-up periods [33,38]. Additional trials oversampling Spanish-speaking youths and using the newly adapted versions of the SSIs are needed to gauge the broader and longer-term utility of Project YES, both within and beyond the San Antonio youth community. Furthermore, implementation challenges will be ongoing for the community. Although the resource is free, raising awareness about the platform poses various challenges. Moving ahead, there is a need to examine pathways for integrating Project YES and similar tools into community settings, including schools, to ensure that youths who might benefit from the tool are aware of it and can access it easily, privately, and independently.

### Conclusions

Overall, the cultural adaptation of Project YES demonstrated an acceptable, accessible, and useful mental health support for English- and Spanish-speaking San Antonio youths. Community-facilitated dissemination of Project YES may have helped lower the dropout rates, relative to recent evaluations of web-based youth-focused SSIs, and reach a more racially and ethnically diverse population, including engaging more males than in prior SSI trials, and a sizable LGBTQ+ youth population. There remains a need for additional work on effective strategies for disseminating and implementing web-based SSI platforms as a community-wide resource. Moving ahead, web-based SSIs may remain a valuable resource and a low-cost, accessible, and youth-centered means for augmenting, complementing, and expanding resource-strapped mental health care systems such as that of San Antonio, Texas.

## Appendix

Multimedia Appendix 1 Stories from San Antonio youths.

## Abbreviations

CIE: careless or invalid effort
IRB: institutional review board
LGBTQ+: lesbian, gay, bisexual, transgender, queer and more
PFS: Program Feedback Scale
SMFQ: Mood and Feelings Questionnaire—Short
SSI: single-session intervention
UTTH: University of Texas Teen Health
YES: Youth Empowerment and Support
