# Supplementary material for: A Digital Single-Session Intervention Platform for Youth Mental Health: Cultural Adaptation, Evaluation, and Dissemination
Source: JMIR Ment Health. 2023 Feb 14;10:e43062. doi: 10.2196/43062 (PMC9975917; doi:10.2196/43062)
Supplement: Multimedia Appendix 1 [file mental_v10i1e43062_app1.docx]

**APPENDIX 1**

**A story from the Spanish translation of Project Personality:**

"Durante mi primer año de la prepa, era nuevo en la escuela. No fue fácil. Y para hacerlo aún peor, algunos chicos decidieron meterse conmigo y hacerme su blanco fácil. Se la pasaban diciéndome cosas muy hirientes, un par de ellos incluso querían pelear conmigo, y decirles a otros que no se juntaron conmigo.

Ser acosado por ellos me hizo sentir triste y me dio miedo. Me preocupaba mucho de lo que los demás pensarían de mí. A veces, pensaba que nunca llegaría tener amigos de verdad. Pero durante todo esto, siempre me repetía a mí mismo que no iba a permanecer así para siempre.

Todo el mundo no está estancado en su forma de actuar; las personas pueden cambiar las cosas que hacen. Con el tiempo, los acosadores pueden cambiar. Pueden volverse más amables o dejar de ser malos. A través de la práctica, pueden cambiar su cerebro y cómo actúan. O simplemente dejar de ser así por madurarse. *Cuando utilicé esta nueva forma de pensar, me sorprendió lo mucho mejor que resultaron las cosas.*”

K. J., 17 years old

**This is the story in English:**

"During my freshman year, I was a new kid to the school. It wasn’t easy. And to make it worse, a couple of kids decided to pick on me and make me into a target. They kept saying really mean things to me. A couple of them even wanted to fight or tell other kids not to hang out with me.

Being picked on made me feel sad and scared. I worried about what other kids would think of me. Sometimes, I thought I might never make real friends.

But during all this, I kept telling myself that it wasn’t going to stay this way forever. Not everybody is stuck being the way they act; people can change the things they do. Over time, bullies can change. They can get nicer, or stop being mean. Through practice, they can change their brain and how they act. Or they just grow out of things. When I used this new way of thinking, I was surprised how much better things turned out to be.”

K. J., 17 years old

**A story from the Spanish translation of the ABC Project:**

"Yo he jugado vóleibol desde los 8 años. Siempre ha sido mi actividad favorita. El año pasado, decidí intentar ser parte del equipo de la escuela. Me mejor amiga fue aceptada, pero yo no. Me sentí muy mal cuando me enteré. Mi amiga fue muy amable al respecto, pero yo estaba muy enojada conmigo misma. Me sentí tonta por haber pensado que podría ser parte del equipo. Después de eso las cosas se pusieron muy difíciles. Yo solo me quería esconder. Pasar tiempo con mi amiga, estar en la escuela -- todo parecía demasiado para mí. No tenía energía para nada. Incluso el vóleibol, dejó de ser divertido. Me sentí muy desanimada en ese tiempo. En ese momento no sabía que estaba pasando.

Recuerdo pensar “¿algún día me volveré a sentir como yo misma?”

**This is the story in English:**

"I’ve played volleyball since I was 8 years old. It’s always been my favorite thing. Last year, I decided to try out for my school’s varsity team. My best friend made it, but I got cut. I felt sick when I found out. My friend was nice about it, but I was just so mad at myself. I felt stupid for thinking I could make it. After that, things got tough. I just wanted to hide. Hanging out with my friend, being at school—it all seemed like too much. I didn’t have energy for anything. Even volleyball stopped being fun. I felt really low during that time. At the time, I didn’t understand what was happening.

**I remember thinking, “will I ever feel like myself again?”**

**A story from the Spanish translation of Project CARE:**

"La presión que yo pongo en mí misma para sacar buenas notas, tener éxito en mis actividades extracurriculares y participar en servicio comunitario para alcanzar mi meta de ser médica puede resultar abrumadora. Me preocupa que, si llego a fallar, todo mi trabajo duro y mi dedicación serían en vano. Me preocupa que podría decepcionar a mi familia, amigos, futuros pacientes y a mí misma.

Para combatir estos pensamientos estresantes que me dicen que yo no soy lo suficientemente buena, encuentro formas de ser amable conmigo misma que funcionan para mí. Me permito dedicar tiempo a leer y salir con mi familia en lugar de sabotearme y encerrarme en mi misma. Sigo poniéndome metas y celebrando pequeñas victorias que espero me darán frutos más adelante. Esta bondad hacia mí misma me ayuda a recordar por qué sigo trabajando tan duro: mi meta es desarrollar las habilidades necesarias para darle el mejor tratamiento a mis futuros pacientes, para ser el mejor ser humano que puedo para mi familia y mi comunidad.

*Ser más amable conmigo misma me ayuda a concentrarme en lo que más me importa tanto ahora como en el futuro.*"

- Laura

**This is the story in English:**

"The pressure I put on myself to make good grades, succeed at extracurricular activities, and participate in community service to reach my goal of becoming a doctor, can feel overwhelming. I worry that if I fail, all my hard work and dedication will be for nothing. I will let down my family, friends, future patients, and myself.

To combat these stressful thoughts telling me I’m not good enough, I find ways to be kind to myself that work for me. I let myself spend time reading and hanging out with my family rather than sabotaging myself. I continue to make goals for myself and celebrating the small wins that will pay off later. This self-kindness reminds me why I continue to work this hard: to build the skills necessary to care for my future patients, my family, and my community.

Being kinder to myself helps me focus on what I care about both now and in the future."

- Laura
